# Chromatin Regulation in Development: Current Understanding and Approaches

**DOI:** 10.1155/2021/8817581

**Published:** 2021-02-02

**Authors:** Zi Hao Zheng, Tsz Wing Sam, YingYing Zeng, Justin Jang Hann Chu, Yuin-Han Loh

**Affiliations:** ^1^Laboratory for Epigenetics, Stem Cells & Cell Therapy, Programme in Stem Cell, Regenerative Medicine and Aging, ASTAR Institute of Molecular and Cell Biology, Singapore 138673; ^2^Department of Physiology, NUS Yong Loo Lin School of Medicine, 2 Medical Drive, MD9, Singapore 117593; ^3^School of Biological Sciences, Nanyang Technological University, Singapore 637551; ^4^Laboratory of Molecular RNA Virology and Antiviral Strategies, Department of Microbiology and Immunology, Yong Loo Lin School of Medicine, National University Health System, National University of Singapore, MD4 Level 5, 5 Science Drive 2, Singapore 117597; ^5^Infectious Diseases Translational Research Program, Yong Loo Lin School of Medicine, National University of Singapore, 10 Medical Drive, Singapore 117597; ^6^Institute of Molecular and Cell Biology, Agency for Science, Technology and Research (ASTAR), 61 Biopolis Drive, Proteos #06-05, Singapore 138673; ^7^NUS Graduate School for Integrative Sciences and Engineering, National University of Singapore, 28 Medical Drive, Singapore 117456; ^8^Department of Biological Sciences, National University of Singapore, Singapore 117543

## Abstract

The regulation of mammalian stem cell fate during differentiation is complex and can be delineated across many levels. At the chromatin level, the replacement of histone variants by chromatin-modifying proteins, enrichment of specific active and repressive histone modifications, long-range gene interactions, and topological changes all play crucial roles in the determination of cell fate. These processes control regulatory elements of critical transcriptional factors, thereby establishing the networks unique to different cell fates and initiate waves of distinctive transcription events. Due to the technical challenges posed by previous methods, it was difficult to decipher the mechanism of cell fate determination at early embryogenesis through chromatin regulation. Recently, single-cell approaches have revolutionised the field of developmental biology, allowing unprecedented insights into chromatin structure and interactions in early lineage segregation events during differentiation. Here, we review the recent technological advancements and how they have furthered our understanding of chromatin regulation during early differentiation events.

## 1. Introduction

During natural development, embryonic stem cells progressively lose their pluripotency and upregulate cell fate specification markers, thereby producing hundreds of different cell types. The ability of a single cell to differentiate and give rise to the whole organism has fascinated biologists for decades. Epigenetic regulation, including histone modifications, histone variant substitutions, maternal factors, DNA methylation, and imprinting, plays a crucial role in the specification and determination of cell fate. Epigenetic factors can change chromosome conformation and the weak interacting forces [1], leading to differential gene expression across cell types. Molecular biology techniques such as fluorescence microscopy and RNA interference have only answered particular aspects of the underlying mechanisms. However, more delicate approaches are required to solve increasingly sophisticated questions in the field. The discoveries of a totipotent subpopulation within mouse embryonic stem cell (mESCs) culture [2], expanded potential stem cells (EPSC) [3, 4], and induced pluripotent stem cells with higher potency [5] have reignited the interest in developing media that are capable of maintaining cells with increased differentiation potential. Studies suggest that such potential is linked to the bivalent chromatin [6, 7] and depletion of inhibitory markers that stabilise the cell fate [8]. The mESCs and primed human ESC (hESCs) are capable of differentiating into the trophoblast lineage upon manipulation [9, 10]. However, it remains unknown whether the transdifferentiation into the trophoblast lineage happens after the transition to the totipotent state [11] or induced directly from the alternate pluripotent state [12]. Recent developments in single-cell technology have allowed us to look deeper into cellular networks involving chromatin state and epigenetic regulators in early embryogenesis [13–15]. These proof of concept studies have showcased the potential of single-cell technology in meeting the needs of the field.

## 2. Single-Cell and Low-Input Techniques

**Figure 1:**
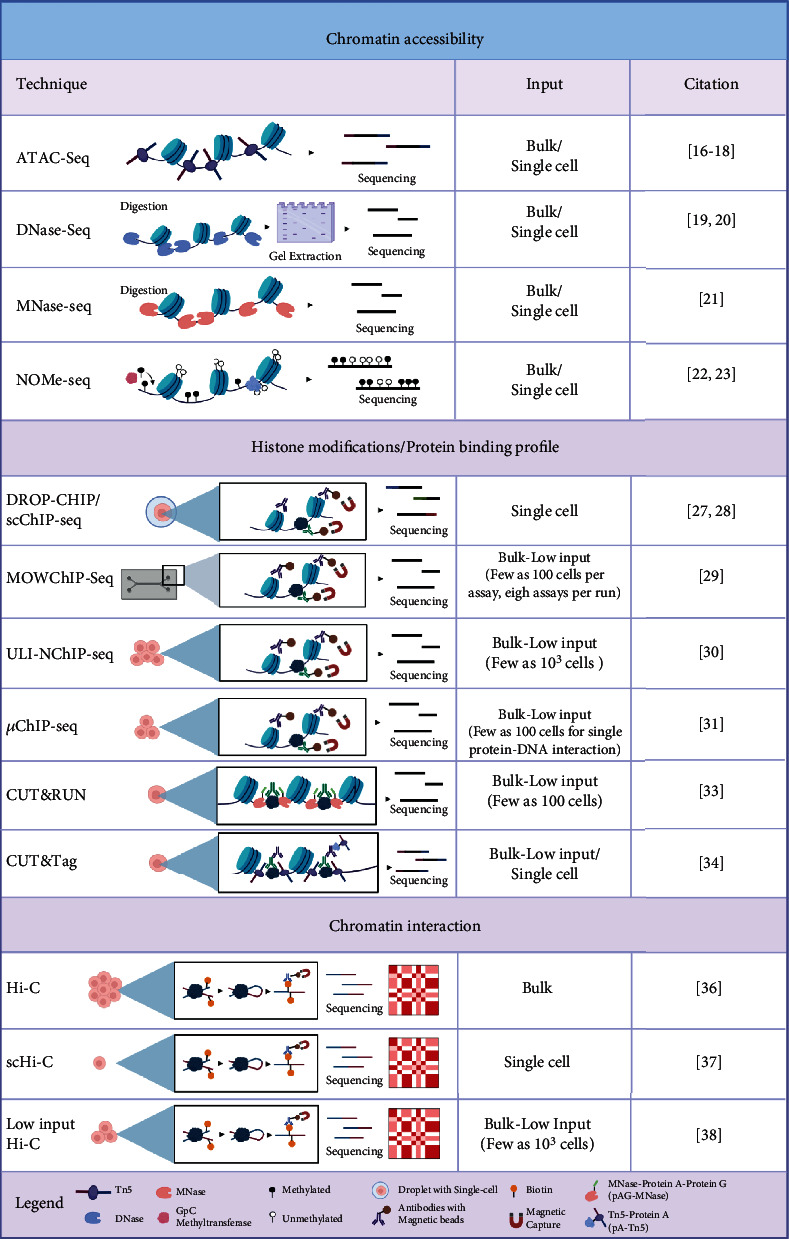
Summary of the comparison of different single-cell and low-input techniques to assess chromatin structure [16–23, 27–31, 33, 34, 36–38]. Created with http://BioRender.com/.

Cellular heterogeneity primes cells towards different lineages and is difficult to study in the context of the embryogenesis. Traditional methods employing the expression of fluorescent proteins and observational studies by perturbing critical factors that are known to be involved in the formation of embryos are both time consuming and inefficient. Additionally, certain cell types with smaller population sizes are easily masked in the bulk analysis. Ever since the advent of single-cell technology in 2009 [16], which permitted the analysis of the mouse embryonic transcriptome, the field has quickly adapted this concept to questions highly relevant to epigenetic regulation. However, these methods remain technically challenging, especially during the process of amplifying signals from each cell while suppressing unspecific noises. Epigenetic studies often involve a bulk analysis of materials pooled together using millions of cells to derive the most accurate map, which is not practical in studies involving early embryos. To this end, various groups have employed different methods, such as multiple rounds of bar coding and specialised beads to improve capturing and accuracy of amplification of the epigenome [14, 17, 18] (Figure 1).

Chromatin accessibility reflects, to some degree, the expression status of genes by controlling the exposure of genomic regions to transcription factors (TFs) and other DNA-binding elements. There are currently four approaches to analyse chromatin accessibility in a single cell. Three of them quantify enrichment of DNA fragments after enzymatic DNA cleavage of accessible regions. The assay for transposase-accessible chromatin using sequencing (ATAC-seq) employs the hyperactive transposase Tn5 which simultaneously cleaves and inserts itself to the accessible regions and ligates sequencing indexes containing adaptors to these regions in each cell (Figure 1). The resultant DNA fragments are amplified *via* polymerase chain reaction (PCR), and short fragments are selected to remove partially digested fragments that are longer in length [19–21]. A second approach employs the so-called DNase I hypersensitive site sequencing (DNase-seq), whereby DNase-sensitive chromatin is cleaved and further processed with either type II restriction enzyme digestion or size selection to obtain fragments with appropriate sizes for sequencing [22, 23]. A third approach is labelled micrococcal nuclease sequencing (MNase-seq), whereby the DNA nuclease digests naked DNA and leaves DNA that binds to the nucleosomes intact, which allows profiling of the inaccessible chromatin in the cell [24]. Lastly, a fourth approach is the “nucleosome occupancy and methylome sequencing” (NOMe-seq), in which a GpC methyltransferase is used to mark accessible regions with GpC methylation (Figure 1). This is followed by bisulfite sequencing of nonmethylated cytosine to obtain information on regions that are not protected by the nucleosomes [25, 26]. Recent advancements in single-cell chromatin accessibility assays involve combinations of multiple readouts to maximize information extracted from the same cell [27–29]. Each method comes with its own bias in enrichment or loss of signals. In addition, these approaches are costly, hence demanding careful consideration before embarking on the experiment.

Chromatin-immunoprecipitation-sequencing (ChIP-seq) is a commonly used technique to examine the interactions between protein and genomic DNA. Incorporating advancements of single-cell technologies, droplet-based single-cell ChIP-seq (DROP-ChIP/scChIP-seq) has since undergone rapid development and has been applied in many studies for understanding the heterogeneity within such cell populations comprehensively [18, 30] (Figure 1). Furthermore, multiple techniques such as the microfluidic-oscillatory-washing-based ChIP-seq (MOWChIP-seq), ultra-low-input native ChIP-seq (ULI-NChIP), and micro-ChIP (*μ*ChIP) have since been developed to overcome challenges that arise from low-input cell numbers and the scarcity of some tissue samples, allowing for high throughput evaluations of cell chromatin status [31–34] (Figure 1). A unique method that fuses an antibody to Tn5, termed CUT&RUN [35] or CUT&Tag [36] (Figure 1), has also opened new avenues in profiling the effects of chromatin remodelling complexes coupled with histone modifications, RNA polymerase II, and TFs in single cells [17].

Chromosome conformation capture or Hi-C is a method that enables the analysis of chromatin interactions (Figure 1). In Hi-C, interacting DNA fragments are ligated and sequenced to detect genome-wide long-range DNA interactions, which provides information on spatial arrangement and proximity of genes and their enhancers. Chromatins are partitioned into self-interacting active and silent topological associated domains (TADs), suggesting a relationship between gene activities and genome folding [37]. However, resolution remains a major issue for single-cell Hi-C over low-input Hi-C [38, 39].

## 3. Roles of Histone Variants on Chromatin Remodelling during Differentiation

**Figure 2:**
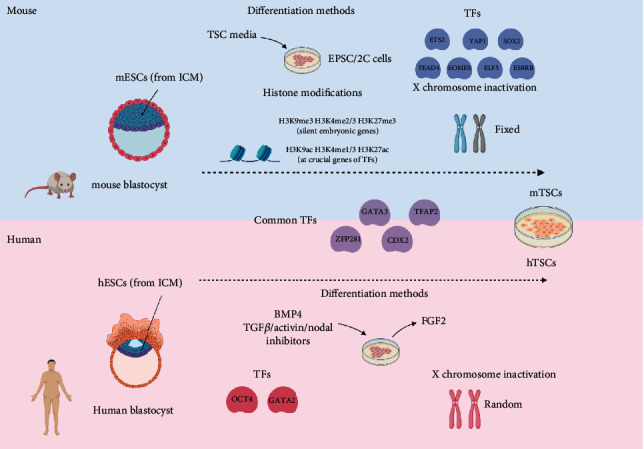
Summary of the comparison in deriving mouse and human ESCs and TSCs from mouse and human ESCs [10, 15, 57, 58, 65, 69–72, 75, 76, 82–85]. Created with http://BioRender.com/.

Extensive rewiring in chromatin regulation, including histone modifications, expression and binding of TFs, and genomic interactions, happens during differentiation. Here, we evaluate the roles of epigenetic factors in chromosome remodelling during differentiation, as well as the differences in the core regulatory network in the transition of human and mouse ESCs to trophoblast stem cells (TSCs) (Figure 2).

Chromatin structure is based on the coiling and positioning of the nucleosome, which is made up of two identical subunits consisting of four histone proteins, H2A, H2B, H3, and H4, while the H1 histone binds to linker DNA. After fusion of the two germ cells into a single zygote, the histone composition undergoes rapid changes to be replaced by newly synthesized canonical histones. It has been implicated that expression of zygotic genes is independent of higher order chromatin structure [40, 41]. Cell fate then appears to be marked as early as the 4-cell stage by the core pluripotent markers [42–44]. During the course of embryogenesis, the chromatin progressively loses its open state, gaining a more condensed conformation.

The roles of noncanonical histones have been widely implicated in stem cell differentiation. In hESCs, depletion of histone 3 variant centromere protein A (CENP-A) has no effect on the self-renewal of stem cells but causes cell cycle arrest at the G1 during differentiation. It also impacts the repair mechanism of the stem cell, leading to apoptosis. Whereas in fibroblasts, depletion of CENP-A leads to increased apoptosis and reduced self-renewal capacity [45]. It remains unknown how centromeres are regulated by CENP-B, CENP-C, and CENP-T during the differentiation and self-maintenance of stem cells.

Investigations into histone variant H3.3 have uncovered its crucial role in differentiation, cell fate transition, and the maintenance of heterochromatin integrity at the centromeres, telomeres, and pericentromeric sites [46]. In particular, the H3.3 lysine 4 residue is associated with enhancers and promoters of active genes, facilitating nucleosome deposition, histone replacement, and binding of chromatin remodelers at those sites [47].

On the other hand, the histone 2 variant H2A.Z is essential in marking genes to be downregulated during differentiation by interacting with polycomb repressive complex 2 (PRC2) to deposit repressive H3K27me3 marks [48]. It is enriched at active enhancers and promoters, affecting the accessibility of the transcription start site to the transcription factors [49, 50]; H2A.Z also interacts with lysine acetylation marks on H3 and CHD4 to remodel chromosomes during stem cell maintenance and differentiation [51, 52].

Each species has its own unique H1 variants serving different functions [53]. There are limited studies in this area, and it is currently thought that H1 controls chromatin compaction by regulating H2AK119ub1 during mESC differentiation [54].

## 4. Histone Modifications

There have been extensive studies on post-translational modifications of Histone 3, which have shown that the pattern of histone modifications is expressed in a lineage-specific manner in the ESC and TSC state. Bivalent marks, namely the active marker H3K4me3 and repressive marker H3K27me3, are unique characteristics in ESCs [6]. These marks poise genes that are expressed when ESCs are committed to lineage specification, and their roles have been studied for a long time. Recent evidence suggests their crucial role in remodelling chromosome accessibility and chromatin looping [55]. However, their specific functions remain largely unknown [56].

Genome-wide analysis performed by Rugg-Gunn et al. suggests that H3K27me3 and H3K9ac levels are higher in the inner cell mass as compared to the trophoblast lineage, although there is no direct evidence to support the causative relationship between the two [57]. Additionally, either trivalent histone marks such as H3K9me3, H3K4me2/3, and H3K27me3 or bivalent histone marks can be adopted in silencing embryonic genes in cells developing into the trophoblast lineage [58] (Figure 2). CDX2 and EOMES are crucial TFs in the establishment of the TSC cell fate and are enriched with active histone marks such as H3K9ac and H3K4me3 while having lower levels of repressive histone marks [57]. In another study, inducing *CDX2* expression resulted in decreases in the expression of pluripotent genes *OCT4* and *NANOG*, increases in trophoblast lineage genes, and the differentiation of TSCs in the mouse embryo [59].

Moreover, it has been reported that histone methyltransferase *SUV39H1* mediated trimethylation on H3K9 is attributed to the suppression of embryonic genes in TSCs [60]; H3K9me3 also interacts with heterochromatin protein 1 to condense and silence different gene sets during differentiation in hESC and mESC [61, 62], highlighting the indispensable role of histone modifications in the regulation of lineage-specific genes.

Enrichment of H4K20me3 during differentiation leads to formation of pericentric heterochromatin by acting with *SMYD5*, and it has been shown that reduced transcription of endogenous long interspersed nuclear elements (LINEs) and long terminal repeats (LTRs) is key in maintaining pluripotency [63].

## 5. Comparison in the Development of Human and Mouse Trophoblast-Related Lineage

In the mouse embryo, specification starts at the 4-cell stage [43], whereas current evidence implies that such specification occurs in the early blastocyst stage in human embryos [64, 65]. In the mouse embryo, implantation is initiated by the mural trophectoderm (TE) followed by the polar TE. In the human embryo, implantation is initiated by the polar TE. The TE layer in human and mouse embryo subsequently matures to give rise to the syncytiotrophoblast (ST) and the extravillous cytotrophoblast (EVT) *via* cell fusion and endoreduplication, respectively. The mouse TE subsequently forms three distinct layers of trophoblast derivatives, separating maternal and fetal blood, whereas in human trophoblast analogs, a different structure is formed with only one layer separating maternal and fetal blood [66]. While there are studies aiming at establishing three-dimensional [67] and two-dimensional trophoblast cultures [68] that each are able to differentiate into both the ST and EVT lineages, there is a lack of studies looking into the role of chromatin remodelling and epigenetic regulation in such models.

The similarities and differences in human and mouse TSCs are well manifested through the aforementioned aspect of physiology. While most of the discussion is focused on the signalling pathway that contributes to the successful differentiation from ESCs to TSCs, the underlying conservativeness in the regulation of chromatin and binding of specific transcription factors is still crucial for the transcriptional network that drives the specification of TSCs.

## 6. Expression of Transcription Factors and Their Binding to Genomic DNA Regions

Transcription factors are known to be bound to specific genes to regulate gene expression directly or indirectly by recruiting other transcription factors (or repressors), or histone modifiers to activate or silence genes. *ZFP281* was identified as a conserved factor critical to the maintenance of human and mouse TSCs. By interacting with MLL and COMPASS subunits and binding to the promoters of target genes, *ZFP281* helps to establish the specific transcriptome necessary for differentiation and specification of mouse TSCs. Moreover, it has been demonstrated that *ZFP281* facilitates the induction of trophoblast stem-like cells from mouse embryonic stem cells upon overexpression. In humans, *ZFP281* helps to stabilise the transcriptome in undifferentiated TSCs [69].

Mouse TSC determination involves genes such as *TEAD4*, *CDX2*, *SOX2*, *ESRRB*, *TFAP2*, *ETS2*, *ELF5*, *GATA3*, and *YAP1* (Figure 2), although it is not known how all these genes interact in this context [10]. On the other hand, a group has recently identified the generation of human induced TSCs through stepwise or direct reprogramming of human dermal fibroblast. TE-associated transcription factors, *TFAP2C* and *GATA2*, are significantly upregulated during reprogramming to naïve state, and supporting their reprogramming to iTSCs [15].

*CDX2* is expressed as early as the 8-cell stage and plays a critical role during the differentiation of cells into the TE and subsequent regulation of TE functions. However, it is not essential for the initiation of TE lineage segregation as *CDX2* knock out embryos retained the ability to form blastocoel cavities, implying that other key genes regulated this process. One such gene could be *TEAD4*, where knockout cells failed to differentiate into the TE, and *TEAD4* knockout embryos were unable to develop into blastocysts [70–72]. While expression of *OCT4* and *CDX2* is critical in the human TE, *OCT4* is depleted in the differentiating mouse TE [73]. Contrastingly, in human embryos, *CDX2* is only expressed after blastocyst formation [74].

*ELF5* has been described as one of the core genes that regulate the self-renewal and differentiation of TSCs. It interacts with EOMES to recruit TFAP2C to TSC-specific genes, thereby inducing their expression in mouse TSCs [75]. Moreover, *Elf5* was found to be methylated and repressed in mESCs but hypomethylated and activated in mTSCs. It promotes the expression of a network of TFs, including *CDX2* and *EOMES*, that drives the efficient differentiation of ESCs to trophoblast-related lineages [65]. The GATA2/3-TFAP2A/C network was enriched in regions of inactive placental and pluripotent genes in hESCs after treatment with BMP4, which induces trophoblast-specific genes and suppresses pluripotency during the initial stage of trophoblast differentiation [76].

Super-enhancers (SEs) are also one of the elements that model the transcriptional network. By mapping these SEs in mTSCs, more than 150 TFs, excluding master TFs such as *CDX2*, *GATA2*, and *TEAD4*, were identified as potential contributors to the TE lineage. This approach opens up a new aspect to further elucidate the mechanisms and regulators of mTSC lineage specification [77]. Additionally, it has been proposed that *ESRRB* could directly regulate the core genes of the TSC self-renewal regulatory network such as *CDX2*, *EOMES*, and *SOX2* [78]. Members of the ERV family RLTR13D5 could also act as enhancers; they are bound by H3K4me1 and H3K27ac, therefore providing binding sites for *CDX2*, *EOMES*, and *ELF5* [79].

## 7. X-Linked Genes

Studies revolving around long noncoding RNA (lncRNA) have shed some light in their roles in regulation of the stem cell pluripotency and lineage segregation. lncRNA recruits chromatin modifiers such as mixed-lineage leukemia 1 (MLL1) and *PRC2* to modulate chromatin structure and gene expression [80, 81]. The study of X-linked genes patterning and X chromosome inactivation by lncRNA X-inactive-specific transcript (XIST) has provided some clues to early developmental events. During lineage segregation in the female mouse embryo, paternal X chromosome is first inactivated, contributing to the TE lineage, followed by reactivation in the inner cell mass (ICM) and finally random X chromosome inactivation. Whereas in the human female embryo, random X chromosome inactivation first occurs in cells contributing to the TE, followed by a second wave of random X chromosome inactivation in ICM [82]. The inactivation is initiated by expression of *XIST* and accompanied by the recruitment of multiple chromatin modifiers to suppress the expression of extra X-linked genes [83]. In contrast to two distinct lineage segregation events in the mouse blastocyst, evidence suggests that the TE, epiblast, and primitive endoderm might arise simultaneously during a single event in human blastocysts [84, 85] (Figure 2).

## 8. Transposable Elements Function in TSC and ESC

Transposable elements account for at least 40% of the human or mouse genomes [86, 87]. Previously regarded as “junk DNA”, it was recently discovered that transposable elements adopt functional roles akin to enhancers, promoters, or insulators, which are essential to gene regulation [88]. Therefore, it is important to explore their regulatory roles in TSCs and ESCs.

**Figure 3:**
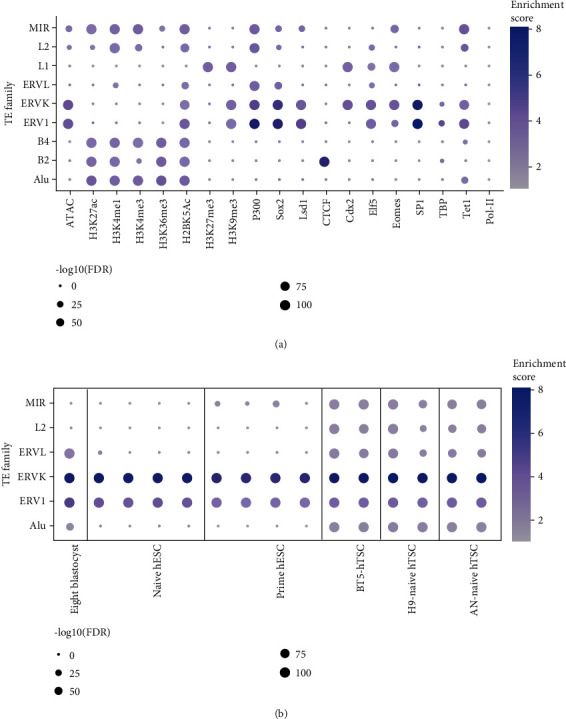
Transposable elements are marked by epigenetic signatures. (a) Dot-plot of the enrichment of transposable elements families in 8 chromatin marks and 11 bound factors in mouse TSCs. The size of the circle represents corrected enrichment *P* values. The colour indicates the enrichment score which was computed with a combination of the binomial test and hypergeometric test [89]. (b) Dot-plot of the enrichment of transposable elements families in open chromatin regions defined by ATAC-seq peaks in human eight-stage blastocysts, naïve ESCs, primed ESCs, blastocyst-derived TSCs, H9-derived TSCs, and AN1 iPSC-derived TSCs. The size of the circle represents corrected enrichment *P* values. The colour indicates the enrichment score which was computed with a combination of the binomial test and hypergeometric test [89].

Transposable elements have contributed greatly to the gene regulatory network in different lineages or cell types [90]. To explore the overall pattern of different epigenetic modifications that accompany transposable elements, we analysed ATAC-seq data [91], histone modification data including H3K27ac, H3K4me1, H3K4me3, H3K27me3, and H3K9me3 [92], H3K36me3 and H4K20me1 [93], H2BK5ac [94], datasets regarding transcription factors such as *P300* [77], *SOX2* [92], *ELF5*, *EOMES*, and *CDX2* [92], *TET1* [95], *CTCF*, *SP1*, and *TBP* [93], and *LSD1* [96] in mouse TSCs. TE family enrichment analysis were done on these data using the same method suggested by the Cao's team [89]. Result (Figure 3(a)) shows that the endogenous retrovirus-like elements (ERVs) such as the ERVK and ERV1 families are significantly enriched in the open regions of mTSCs and bound by critical TSC-related TFs. Furthermore, transposable elements such as B2, Alu, and MIR (Mammalian-wide interspersed repeats) are bound by active histone marks such as H3K4me1 and H3K27ac, implying possible functions as enhancers. Promoters are conserved across species, whereas enhancers are found to be specific to different organisms or cell types. As enhancers are known to regulate tissue- or cell type-specific gene expression, we overlapped the TE sites with enhancers defined by P300 and H3K27ac. The TE-derived enhancers such as ERVK and ERV1 were significantly bound by transcription factors *SOX2*, *LSD1*, *EOMES*, and *ELF5*. Given the functions of the factors discussed in earlier sections, the analysis suggests that these repeats could act as enhancers to regulate gene expression in TSCs. RLTR13D5 containing ERVK-derived enhancers echoes the significance of ERV in the mTSCs by acting as enhancers and binding sites for TSC-specifying TFs [79, 97].

Understanding the conservation of chromatin accessibility across hTSCs and mTSCs might provide novel insights into their differences. To this end, we analysed ATAC-seq datasets from naïve hESCs, primed hESCs, blastocyst-derived TSCs, and naïve hESCs [98, 99]. The TE families enrichment analysis shows that ERVK and ERV1 were significantly enriched in hESCs and hTSCs (Figure 3(b)), suggesting that ERVK might play conserved and functional roles in TSCs in both species. There are also both unique open ERVKs and shared open ERVKs in ESC and TSC. From the motif analysis, ERVKs with open chromatin state in hTSCs are enriched for TSC-related transcription factor motifs such as *TEAD4* and *GATA3*, suggesting that these ERVKs might have been adopted during evolution to cooperate with TSC-specific transcription factors to regulate transcriptional networks essential for TSC.

Apart from expression of TFs and chromatin accessibilities, recent Hi-C data has revealed the divergence in the repressive and active chromatin interaction between mouse ESCs and TSCs lineages. TSCs genes, which are repressed in ESCs, interact with H3K27me3 associated regions in ESCs through the PRC. Furthermore, enhancer-gene interactions involving key TSC transcription factors are particularly enriched to maintain the expression of TSC-genes [100]. Another recent report correlates the chromatin modifier known as the ChAHP complex (CHD4, ADNP, and HP1) with proper cell differentiation. This complex competes with CTCF binding sites and modulates the formation of TADs in proximal regions, specifically at conserved SINE B2-transposable elements [101]. The role of the ChAHP subunits CHD4 and HP1 in stem cell maintenance and differentiation has been previously reported [51, 61].

To identify the target genes regulated by transposable elements, Hi-C or promoter capture techniques could be used to check the putative targets of these TE-derived enhancers. CRISPR interference methods could be used to disrupt the transposable elements followed by validation using RNA-seq or qPCR analysis to check the expression of the putative target genes. As demonstrated by Todd et al. [97], only small subsets of transposable elements are crucial in regulating the TSC and ESC gene expression. Therefore, it is important to pinpoint those that have been adopted as functional gene regulatory elements during evolution critical in each cell type.

## 9. Future Perspective

There are numerous pluripotent states reported in human and mouse ESCs, with the most common ones being the naïve and primed state. There are a multitude of studies that attempted to differentiate primed hESC from TSC with varying scales of success [10]. It was reported that during differentiation of human ESCs towards TSCs, FGF2 should be removed completely from the media, and BMP4 and TGF*β*/activin/nodal inhibitors should be added as supplements. The size of the initial colonies also affects the outcome of the differentiation process. Meanwhile, 2C-like cells [2] and EPSCs [4] are the only two reported sources of mouse stem cells that are capable of differentiating into TSC *in vitro*, making it worthwhile to dissect the mechanism underlying the derivation of TSCs in the respective state.

It has been established that hESCs exist in the primed pluripotent state with one active X chromosome and one inactive X chromosome. This resembles a closer gene expression and signaling profile to primed mouse epiblast stem cells (mEpiSCs) than mouse ESCs, which is considered an earlier stage of naïve pluripotent state with two active X chromosomes [102, 103]. In humans, naïve pluripotent stem cells express TFs and display open chromatin structure associated with cells from trophoblast-related lineages, which were conversely reported to be able to give rise to self-renewing TSCs, a feat which is unachievable with primed hESCs that are exposed to the same differentiation assays [98, 104]. Similar phenomena were observed in the mouse, when overexpression of *CDX2* in the naïve mESCs drove the cells towards a TSC-like cell fate, but not mEpiSCs [105].

Early studies characterizing hESC-derived trophoblast-like cells focused on human chorionic gonadotropin production and cellular invasion capacity. While some studies claim that mouse or human TSCs derived *in vitro* closely resemble their *in vivo* counterparts, others have provided contradictory results [12, 98]. This might be due to differences in the parameters used by each group during cell type characterization and culturing, as studies have shown that differences in starting colony and chemical providers could drastically alter the results [10]. It will be interesting to apply novel single-cell technologies to improve the characterization and understanding of cellular heterogeneity and help to reconstruct a clearer picture of cellular processes, including chromatin remodelling events during changes in the cell fate.

## 10. Conclusion

In the last three decades, a considerable amount of effort has been invested to our understanding and capturing cells in different pluripotency states ranging from TSCs, expended potential, 2C-like, naïve, prime, Rosette [106], Founder [107], and many more. Researchers have employed a wide range of methods to delineate their differences and analogues *in vivo* and across different animal species. While single-cell RNA-seq datasets have provided insights into the transcriptome of different cell types and revealed details on rare populations and the trajectory of cells during differentiation, this information is often limited and does not provide sufficient data to derive the factors and mechanisms controlling the specification and determination of each cell type.

Although pluripotency circuitry has been well studied, novel stem cell populations and pluripotent stages are consistently being reported. The ability of cells to form blastocyst-like structures [108] to investigate cell fate changes *ex vivo* has recently gained vast interest. Transposable elements, previously disregarded as an unimportant part of the genome, proved to be essential in controlling totipotency in the mouse, while showing differentially binding to pluripotent and TSC-specifying genes. There is still a broad gap in knowledge regarding the epigenome within each cell in early embryogenesis, priming them to different fates under the same condition.
